# Case report: A familial B-acute lymphoblastic leukemia associated with a new germline pathogenic variant in *PAX5*. The first report in Mexico

**DOI:** 10.3389/fonc.2024.1355335

**Published:** 2024-03-20

**Authors:** Joaquín García-Solorio, Octavio Martínez-Villegas, Ulises Rodríguez-Corona, Carolina Molina-Garay, Marco Jiménez-Olivares, Karol Carrillo-Sanchez, Elvia C. Mendoza-Caamal, Anallely Muñoz-Rivas, Beatriz E. Villegas-Torres, Alejandra Cervera, Luis L. Flores-Lagunes, Carmen Alaez-Verson

**Affiliations:** ^1^ Laboratorio de Diagnóstico Genómico, Instituto Nacional de Medicina Genómica (INMEGEN), Mexico City, Mexico; ^2^ Departamento de hemato-oncología pediátrica, Unidad Médicade Alta Especialidad Hospital de Ginecología Pediatría No 48, Centro Médico del Bajío, León, Guanajuato, Mexico; ^3^ Ribonucleo Protein Biochemistry Research Unit, Montreal Clinical Research Institute, Montreal, QC, Canada; ^4^ Subdirección de Genómica Poblacional, Instituto Nacional de Medicina Genómica (INMEGEN), Mexico City, Mexico

**Keywords:** acute lymphoblastic leukemia, *PAX5*, germline variant, leukemia predisposition, WES

## Abstract

B-cell acute lymphoblastic leukemia (B-ALL) is one of the most common childhood cancers worldwide. Although most cases are sporadic, some familial forms, inherited as autosomal dominant traits with incomplete penetrance, have been described over the last few years. Germline pathogenic variants in transcription factors such as *PAX5, IKZF1*, and *ETV6* have been identified as causal in familial forms. The proband was a 7-year-old Mexican girl diagnosed with high-risk B-ALL at five years and 11 months of age. Family history showed that the proband’s mother had high-risk B-ALL at 16 months of age. She received chemotherapy and was discharged at nine years of age without any evidence of recurrence of leukemia. The proband’s father was outside the family nucleus, but no history of leukemia or cancer was present up to the last contact with the mother. We performed exome sequencing on the proband and the proband’s mother and identified the *PAX5* variant NM_016734.3:c.963del: p.(Ala322LeufsTer11), located in the transactivation domain of the PAX5 protein. The variant was classified as probably pathogenic according to the ACMG criteria. To the best of our knowledge, this is the first Mexican family with an inherited increased risk of childhood B-ALL caused by a novel germline pathogenic variant of *PAX5*. Identifying individuals with a hereditary predisposition to cancer is essential for modern oncological practice. Individuals at high risk of leukemia would benefit from hematopoietic stem cell transplantation, but family members carrying the pathogenic variant should be excluded as hematopoietic stem cell donors.

## 1 Introduction

B-cell acute lymphoblastic leukemia (B-ALL) is the most common cancer in children. Several genetic alterations acquired by leukemic cells have been recurrently identified, and some have been used as biomarkers for disease classification, risk stratification, and treatment selection. The increased use of genomic sequencing methodologies in the study of familial and sporadic ALL cases has led to the identification of novel genetic predisposition syndromes associated with a high risk of ALL development. Germline pathogenic variants in genes such as *PAX5, IKZF1*, and *ETV6* increase the risk of B-ALL, which is sometimes accompanied by chronic symptoms such as thrombocytopenia in ETV6-related diseases. In most cases, the disease exhibits an autosomal dominant pattern with incomplete penetrance (1). Additionally, inherited cancer susceptibility syndromes, such as Li-Fraumeni, and some genetic conditions also increase the risk of hematological malignancies in children (2).

Here, we report a case where the proband and her mother developed B-cell precursor ALL during childhood. Exome sequencing revealed a new germline loss-of-function pathogenic variant of *PAX5* in both affected individuals.

## 2 Case presentation

A 7-year-old Mexican girl diagnosed with B-ALL was referred to the Genomic Diagnostic Laboratory of the National Institute of Genomic Medicine (INMEGEN). B-ALL diagnosis was established at five years and 11 months of age. The initial complete blood count showed leukocytosis of 20,670 cells/μL with 85% blasts, hemoglobin concentration of 6 g/dL, hematocrit of 18%, mean corpuscular volume of 97 fl, and platelet count of 14,000/μL. Bone marrow biopsy showed diffuse infiltration with 98% blasts and L1 morphology of the French-American-British (FAB) classification. Immunophenotyping revealed expression of CD45, CD34, CD19, CD58, CD10, CD22, CD24, CD123, and CD81. The conventional cytogenetic study indicated hypodiploidy (35-40 chromosomes); therefore, her leukemia was classified as very high-risk (the clinical features are summarized in **Table 1**). The proband underwent chemotherapy and was enrolled in the 02-16 protocol from the National Medical Center, “La Raza”, adapted from the Dana-Farber ALL Consortium Protocol 11-001. Complete remission was achieved, and minimal residual disease on day 28 was negative by flow cytometry. Central nervous system prophylaxis was scheduled, and intensification began one month later. The patient is currently under surveillance with an event-free survival of 60 months. The last update from the patient’s mother was in January 2024. She said her daughter is doing well, is a third-grader in elementary school, and has normal academic achievement.

**Table 1:** Summary of the proband’s clinical features.

Data	Patient information
Sex	Female
Age at diagnosis	5 years, 11 month
Risk stratification	High risk
White blood cell count at diagnosis	20,670 cells/μL
Bone marrow blast cells at diagnosis	85%
Karyotype	Hypodiploidy
Immunophenotype	CD45, CD34, CD19, CD58, CD10, CD22, CD24, CD123, and CD81
French-American-British (FAB) classification	L1
MRD on day 28	Negative
Family history of B-ALL	Yes (Mother)
Family history of other malignant neoplasms	No reported

Family history showed that the proband’s mother had high-risk B-ALL at 16 months of age. She received chemotherapy and was discharged at the age of nine. There has been no evidence of recurrence since then. At the time of this study, she was 26 years old. A board-certified clinical geneticist explored the proband and her mother, observing no dysmorphic or other relevant clinical features. No thrombocytopenia or qualitative abnormalities affecting platelet function were identified during the surveillance period. The proband’s father was outside the family nucleus, but no history of leukemia or cancer was present up to the last contact with the mother. Data on paternal grandparents are unknown. Based on these findings, we performed exome sequencing for both individuals.

## 3 Material and methods

Informed consent for the molecular study and its publication was obtained from the mother. Genomic DNA (gDNA) was extracted from the peripheral blood with a Maxwell^®^ 16 Blood DNA Purification Kit (*Promega, Madison, WI, USA*). The purity and concentration of the DNA samples were measured using a NanoDrop 1000 spectrophotometer (*Thermo Fisher Scientific, Waltham, MA, USA*) and Qubit fluorometer (*Thermo Fisher Scientific, Waltham, MA, USA*). Library preparation for next-generation sequencing (NGS) was conducted following the manufacturer’s protocol with the “Whole Exome Sequencing Panel Kit” version 1 (*Sophia Genetics SA, Saint Sulpice, Switzerland*). After library quality control, sequencing was performed using a NextSeq 500 instrument (*Illumina, San Diego, CA*, USA). Sequencing data analysis and variant annotation were carried out using the Sophia DDM^®^ software (*Sophia Genetics SA, Saint Sulpice, Switzerland*). Bioinformatic filters were built by selecting genes related to cancer predisposition syndromes and those conferring risk for the development of hematologic neoplasms.

## 4 Results

**Figure 1:**
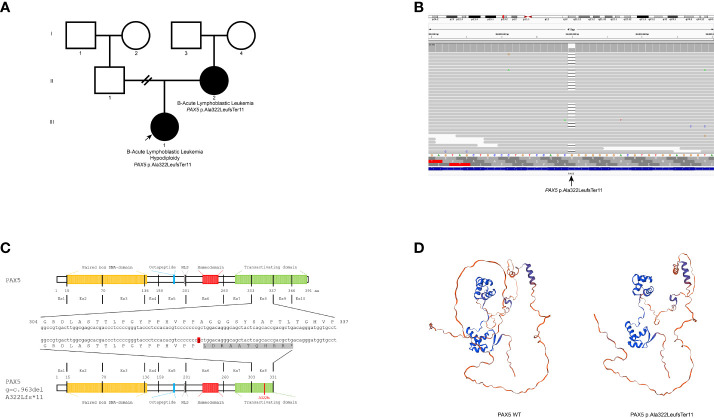
**(A)** Pedigree showing the inheritance of the variant p.(Ala322LeufsTer11) in *PAX5*. **(B)** The aligned NGS sequence reads viewed at the Integrative Genomics Viewer (IGV) showing the variant. **(C)** *PAX5* gene and protein domain structural representation of the nucleotide/amino acid sequence. The mutation p.(Ala322LeufsTer11) is represented in the transactivation domain (green) and the novel ten amino acids due to frameshift alteration (gray). The affected nucleotide is shown in red. **(D)** AlphaFold (3) prediction of the PAX5 wild-type (left) and PAX5 p. (Ala322LeufsTer11) mutated protein (right) by SWISS-MODEL (4).

The heterozygous *PAX5* variant NM_016734.3:c.963del: p.(Ala322LeufsTer11) was identified in both the proband and the proband’s mother (**Figures 1A, B**). No additional pathogenic variants were identified in predisposition syndrome genes or other genes that confer a risk for hematologic neoplasms. The variant p.(Ala322LeufsTer11) was predicted to be likely pathogenic according to the American College of Medical Genetics and Genomics (ACMG) criteria (PVS1, PMS2, and PP1) (5). The one-base deletion resulted in a frameshift and a premature stop codon (**Figure 1C**). The MutationTaster algorithm predicts nonsense-mediated decay (NMD) for this variant. According to the exomes of gnomAD, its population frequency is very low (f=0.000037), with the variant identified in only nine Asian and European individuals out of 242,998. It is absent from the gnomAD genomes and 470 Mexican individuals from the Mexican database. This database is available on the *Franklin by Genoox* platform (as of October 2023).

## 5 Discussion

The impaired activity of critical hematopoietic transcription factors due to inherited germline pathogenic variants has been identified as the cause of some monogenic familial leukemia syndromes. In all cases, susceptibility to B-ALL was inherited as autosomal dominant, with incomplete penetrance.

PAX5 belongs to the PAX family of transcription factors. It has been associated with the development of the central nervous system, B-lymphocytes, and spermatogenesis (6). PAX5 is essential for normal B-cell development and maintenance of B-cell lineage identity. Its expression starts at the pre-pro-B cell stage, continues through pre-B and mature B cells, and stops with final B-cell differentiation (7). *PAX5* is one of the most frequently mutated genes in sporadic B-ALL cases. The frequency of somatic mutations ranges between 25-35%, with most mutations located in the paired box DNA domain or the transactivation domain of the protein (8). The somatic mutational spectrum of *PAX5* involves point mutations, indels, copy number alterations (focal-broader deletions or intragenic amplifications), and translocation, resulting in reduced protein levels of PAX5, hypomorphic alleles, or fusion proteins (9).

In addition to somatic mutations, germline pathogenic variants of *PAX5* have been reported to confer genetic susceptibility to B-ALL development and functioning as essential drivers of leukemogenesis in a few families. Shah et al. (8) described a germline *PAX5* variant, c.547G>A, p.(Gly183Ser), which affects the octapeptide domain, reduces transcriptional activity, and confers susceptibility to B-ALL. This variant was identified by exome sequencing in two families of Puerto Rican and African American ancestry (8). Three members of a Jewish family with leukemia also carried a p.(Gly183Ser) germline variant and somatic structural modification of the 9p region, which resulted in the loss of the wild-type *PAX5* allele (10). In 2022 another family with c.547G>C p.(Gly183Arg) was reported (11).

The germline pathogenic variant c.113G>A, p.(Arg38His) was identified in three family members with B-ALL, aged between 11 and 25 years, whose parents were asymptomatic (1). The identified mutation was heterozygous and predicted to affect the affinity of PAX5 for DNA (12). Interestingly, in two of the three members, the p.(Arg38His) variant co-occurred with the somatic p.(Arg140Leu) *PAX5* variant (1). A significant difference in the age at onset of leukemia between patients with p.(Arg38His) and p.(Gly183Arg) was observed. It suggests a higher penetrance for p.(Gly183Arg) (60-62%) than p.(Arg38His) (36.4%) (11). Moreover, thrombocytopenia or bleeding disorders associated with other hematological neoplasm predisposition syndromes have not been found in patients with *PAX5* germline mutations (11). Quantitative or qualitative platelet abnormalities seem to be restricted to patients with germline defects in the transcription factors required to regulate megakaryopoiesis and platelet production, such as RUNX1, GATA-1, FLI1, GFI1b, and ETV6 (13).

The variant p.(Ala322LeufsTer11) has not been reported as a germline pathogenic variant in the ClinVar database or in the professional version of the Human Genome Mutation Database (as of October 2023). It is the first germline frameshift variant of *PAX5* that increases the risk of B-ALL. Germline variants causing loss-of-function are infrequent in *PAX5*; a significant constraint for loss-of-function variants is observed in the gnomAD database (pLI = 1, o/e = 0 (0–0.17)), based on calculations using the canonical transcript (ENST00000358127.4) (14).

To identify whether this variant is present in Mexican pediatric patients with sporadic B-ALL, we reviewed NGS-identified *PAX5* mutations in 206 Mexican Mestizo *de novo* B-ALL patients analyzed in our laboratory (the manuscript was already accepted in Frontiers Oncology Number 1337954). None carried p.(Ala322LeufsTer11), and three cases had a different variant in the same position: c.963dup, p.(Ala322ArgfsTer19). The variant allele fraction (VAF) ranged between 25 and 27%, suggesting a somatic origin in all three cases. None of the patients had a second *PAX5* allele mutated or deleted. Gu et al. also identified nine patients with *PAX5*: p. A322fs in a sample of 1988 childhood and adult B-ALL cases. The VAF in these cases was also suggestive of a somatic origin (15). According to the Catalogue of Somatic Mutations in Cancer (COSMIC database), p.(Ala322LeufsTer11) has been identified in eight patients with solid tumors, including endometrioid carcinoma (16), adenocarcinoma, renal cell carcinoma (17), and ductal carcinoma (18). This suggests that Ala322 is recurrently mutated in cancer and supports an oncogenic role for this mutation.

The p.(Ala322LeufsTer11) variant resulted in the loss of 15% of the sequence (60 amino acids) from the transactivation domain located at the carboxylic end of the protein. NMD was predicted to occur, causing loss of function. However, if NMD does not happen, an abnormal PAX5 protein would be produced with a truncated transactivation domain and a tail of ten amino acids that does not correspond to the wild-type PAX5 protein. This protein would probably be non-functional (**Figure 1D**). Van Engelen et al. (19) described a patient with a germline exon 6 deletion in *PAX5* that caused a frameshift with a premature stop in exon 7. The variant predicts NMD or results in a truncated protein with loss of C-terminal transactivation and inhibitory domains, similar to our variant. These authors demonstrated equivalent expression levels of transcripts lacking exon 6 to those of the wild-type allele, suggesting that NMD may not occur. The expression of truncated PAX5 proteins, originating from in-frame and out-of-frame deletions, has been shown to cause loss of function.

*PAX5* has been identified as a candidate gene for autism spectrum disorder (ASD) in several sequencing studies (20–22). *PAX5* haploinsufficiency was recently associated with neurodevelopmental disorders, including intellectual disability (ID), developmental delay (DD), and ASD. No structural congenital disabilities, dysmorphic features, or recurrent patterns of brain MRI findings were observed in these studies (23), and neither our proband nor her mother presented with these neurological disorders. Additionally, ASD, DD, and ID have not been reported in families with leukemia. Moreover, leukemia did not develop in patients with *PAX5* mutations, as reported by Gofin Y et al. (23).

Other transcription factors, such as *ADNP, FOXP1, TCF7L2*, and *TBLXR1*, have been genetically implicated in autism and cancer (24). It is not understood why *PAX5* mutations cause neurological alterations in some patients while shifting the balance toward leukemia in others. It does not seem to be related to the type of mutation or the affected PAX5 protein domain. Mutations found in patients with neurodevelopmental disorders included whole gene deletions, *de novo* frameshift predicted to cause non-sense mediated mRNA decay, stop-gain mutations located in the transactivation domain that probably generate a truncated protein, and rare missense variants inside of the conserved paired domain or in the transactivation domain (22). However, the number of identified patients with leukemia or neurodevelopmental disorders due to *PAX5* germline mutations is too small to draw definitive conclusions about genotype/phenotype correlations. A better understanding of the role of PAX5 in cell biology and disease is needed to clarify the mechanisms leading to each clinical outcome.

The contribution of germline syndromes to leukemia susceptibility could be underestimated because they are not regularly explored during routine diagnostic algorithms; at least a family history suggestive of leukemia or hematologic abnormalities would prompt suspicions. In the present case, the B-ALL history in the early childhood of the proband and proband’s mother was compatible with germline transmission of the disease. Exome sequencing identified a new *PAX5* germline loss-of-function variant in both individuals. However, risk alleles may be present in patients without clinical findings, suggesting hereditary hematologic malignancy syndromes. Guijarro F et al. (25) identified pathogenic or likely pathogenic variants in *ATM, DDX41, CHEK2, FANCA, FANCM, SBDS, DNAJC21*, and *CSF3R* in 288 acute myeloid patients without criteria that would suggest the presence of hereditary hematologic malignancy syndromes. The existence of *de novo* or low-penetrance variants, late-onset disease, and incomplete family history contributes to a lack of clinical suspicion (26). Additional research is required to evaluate the contribution of these and other germline pathogenic variants to the risk of hematologic neoplasms in children and adults. Recognizing hereditary predisposition in pediatric and adult cancer patients is crucial for early detection, prevention, treatment, and management of associated health risks to improve outcomes for affected individuals and their families. Additionally, in patients with a hereditary predisposition to hematologic neoplasms, searching for the pathogenic variant in potentially related donors should be performed if a bone marrow transplant is considered.

Raising awareness of neoplastic predisposition syndromes and a more comprehensive understanding of inherited genetic risks in patients with leukemia are needed. Including predisposition genes for hematologic neoplasms and solid tumors in NGS multigene panels used for tumor profiling and germline/tumor testing could contribute to achieving this goal. The advantages, disadvantages, and ethical considerations of adopting universal testing for hematologic neoplasms must be carefully evaluated in each clinical and socioeconomic context (27).

## Data availability statement

The datasets presented in this study can be found in the online repository: National Center for Biotechnology Information. ClinVar; [VCV002637935.1].

## Ethics statement

The study was conducted in accordance with the local legislation and institutional requirements. Written informed consent was obtained from the minor(s)' legal guardian for the publication of any potentially identifiable images or data included in this article. All analyses were performed in accordance with the principles of the Declaration of Helsinki.

## Author contributions

JG-S: Conceptualization, Data curation, Formal Analysis, Investigation, Methodology, Resources, Software, Supervision, Validation, Visualization, Writing – original draft, Writing – review & editing. OM-V: Conceptualization, Data curation, Formal Analysis, Investigation, Writing – review & editing. UR-C: Data curation, Formal Analysis, Investigation, Writing – review & editing. CM-G: Methodology, Supervision, Validation, Writing – review & editing. MJ-O: Methodology, Supervision, Validation, Writing – review & editing. KC-S: Methodology, Supervision, Validation, Writing – review & editing. EM-C: Supervision, Writing – review & editing. AMR: Methodology, Supervision, Writing – review & editing. BV: Methodology, Supervision, Writing – review & editing. AC: Formal Analysis, Software, Writing – review & editing. LF-L: Supervision, Validation, Writing – review & editing. CA-V: Conceptualization, Data curation, Formal Analysis, Funding acquisition, Investigation, Methodology, Project administration, Resources, Software, Supervision, Validation, Visualization, Writing – original draft, Writing – review & editing.
